# Immunoinformatic Analysis to Identify Proteins to Be Used as Potential Targets to Control Bovine Anaplasmosis

**DOI:** 10.1155/2020/8882031

**Published:** 2020-08-27

**Authors:** Sergio D. Rodríguez-Camarillo, Rosa E. Quiroz-Castañeda, Hugo Aguilar-Díaz, José E. Vara-Pastrana, Diego Pescador-Pérez, Itzel Amaro-Estrada, Fernando Martínez-Ocampo

**Affiliations:** ^1^Unidad de Anaplasmosis, Centro Nacional de Investigación Disciplinaria en Salud Animal e Inocuidad, INIFAP, C.P. 62574, Jiutepec, Morelos, Mexico; ^2^Unidad de Artropodología, Centro Nacional de Investigación Disciplinaria en Salud Animal e Inocuidad, INIFAP, C.P. 62574, Jiutepec, Morelos, Mexico; ^3^Universidad Politécnica del Estado de Morelos, Paseo Cuauhnahuac 566, Lomas Del Texcal, C.P. 62574, Jiutepec, Morelos, Mexico; ^4^Laboratorio de Estudios Ecogenómicos, Centro de Investigación en Biotecnología, Universidad Autónoma del Estado de Morelos, C.P. 62209, Cuernavaca, Morelos, Mexico

## Abstract

Omics sciences and new technologies to sequence full genomes provide valuable data that are revealed only after detailed bioinformatic analysis is performed. In this work, we analyzed the genomes of seven Mexican *Anaplasma marginale* strains and the data from a transcriptome analysis of the tick *Rhipicephalus microplus*. The aim of this analysis was to identify protein sequences with predicted features to be used as potential targets to control the bacteria or tick-vector transmission. We chose three amino acid sequences different to all proteins previously reported in *A. marginale* that have been used as potential vaccine candidates, and also, we report, for the first time, the presence of a peroxinectin protein sequence in the transcriptome of *R. microplus*, a protein associated with the immune response of ticks. The bioinformatics analyses revealed the presence of B-cell epitopes in all the amino acid sequences chosen, which opens the way for their likely use as single or arranged peptides to develop new strategies for the control and prevention of bovine anaplasmosis transmitted by ticks.

## 1. Introduction

Ticks and tick-borne pathogens constitute a major challenge for the cattle industry due to their impact on production losses [1]. Bovine anaplasmosis is a disease caused by *Anaplasma marginale,* an important tick-transmitted, intraerythrocytic Gram-negative bacterium, that is endemic in Mexico [2]. The disease has a worldwide distribution and causes serious economic losses, particularly in beef cattle, as they are more exposed to *A. marginale* transmitted by the tick-vector *Rhipicephalus microplus* [3]. The control of bovine anaplasmosis does not only depend on controlling the pathogen itself, but also the vector which transmits it as they have coevolved with the host [4].

So far, there are no commercial vaccines against bovine anaplasmosis, and those that have been prepared from freed bacteria (initial bodies) have a limited use due to a wide antigenic diversity of the pathogen, while those prepared from live attenuated organisms carry the risk for the cotransmission of other blood-borne pathogens [2, 5]. With regards to tick vaccination, recent studies have shown that antitick recombinant vaccines such as aquaporin, subolesin, and tick gut glycoprotein Bm86 have a synergistic effect in reducing the engorgement of tick larvae *in vitro* [6, 7, 8]. So, an integral view of the tick-pathogen relationship should be considered to propose not only effective control measures but also successful diagnostic and vaccination methods as those recently reported [8, 9, 10].

Recently, the Next-Generation Sequencing (NGS) in veterinary medicine has revealed the potential to design primary diagnostic, control, and prevention methods [11]. The genomes of *A. marginale* and *R. microplus* have been sequenced and published [12, 13, 14], and many detailed studies have been performed with the Major Surface Proteins (MSPs) of *A. marginale,* whether as vaccine prospects or diagnostic targets. In the first instance, individual or conglomerate proteins have not been very successful inducing protective solid immunity; as diagnostic targets, a recombinant Msp5 has been successfully used for molecular detection and the serological ELISA test [15, 16].

In contrast, in *R. microplus,* several proteins have been studied as vaccine candidates [17]. The use of Bm86 in the vaccines TickGard [18] (no longer commercially available) and GAVAC [19] reduced parasitism poorly, and the immunologic memory induced was short-lived [20, 21]. The recent approaches in tick research have facilitated a different design of vaccines based on genomics, proteomics (hemolymph) [22], and transcriptomics (sialomes) studies [23].

Despite the amount of data produced for both *A. marginale* and *R. microplus,* a wide repertoire of proteins still remains to be studied, as well as the possibility to find protective antigens to bovine anaplasmosis and its vector *R. microplus.*

In this work, we carried out an immunomic analysis of the genomes of all reported Mexican *A. marginale* strains with the aim to find previously nonreported potential vaccine candidates. Derived from a transcriptomic analysis of *R. microplus,* we found a protein involved in immunological processes whose absence makes ticks susceptible to acaropathogenic organisms used in biological control.

## 2. Materials and Methods

### 2.1. Genomic Analysis and Protein Selection

The amino acid sequences for the reported genomes of the seven Mexican strains were searched online for outer membrane and membrane proteins. The sequences were downloaded and grouped in a list, with the exception of the MSPs and the type four secretion system (VirB) sequences.

The sequence of the peroxinectin (*pxn*) gene was searched and retrieved from the transcriptome (unpublished data) of different stages of *R. microplus* (Arthropodology Unit, INIFAP, Mexico). The coding sequence of the *pxn* gene was translated to a protein sequence with the Translate Tool from Expasy.

This study was approved by the Animal Experimentation and Ethics Committee of the National Center for Disciplinary Research in Animal Health and Safety (CENID-SAI, Mexico) which is a branch of the INIFAP. The study took ethical and methodological aspects into considerations in accordance with the Mexican regulations on use, housing, and transportation of experimental animals (NOM-062-ZOO-1999 and NOM-051-ZOO-1995).

### 2.2. Prediction of Antigenic Proteins

To find the highest antigenic protein, selected protein sequences of *A. marginale* and the protein sequence of the peroxinectin of *R. microplus* were submitted to VaxiJen v2.0 server (http://www.ddgpharmfac.net/vaxijen/VaxiJen/VaxiJen.html) with default parameters.

### 2.3. Prediction of Subcellular Localization and Stability of the Proteins

Predicted antigenic proteins of *A. marginale* and the peroxinectin protein of *R. microplus* were submitted to different servers to predict their subcellular localization. We used the secondary structure and subcellular prediction server Constrained Consensus TOPology (CCTOP; http://cctop.enzim.ttk.mta.hu). The proteins of interest were also submitted to the CELLO v.2.5 server (http://cello.life.nctu.edu.tw/). To analyze the stability and secondary structure of the target proteins sequence, ProtParam server (http://web.expasy.org/protparam/) and SOPMA server (https://npsa-prabi.ibcp.fr/cgi-bin/npsa_automat.pl?page = /NPSA/npsa_sopma.html) were used with default parameters.

### 2.4. Linear B-Cell Epitope Prediction

B-cell epitopes can be categorized as linear (continuous) and conformational (discontinuous) based on their spatial structure. We used, at least, three online available tools for the prediction of linear epitopes: ABCpred (http://crdd.osdd.net/raghava/abcpred/) was set at sequences for relevant linear B-cell epitopes at 18-mers with a threshold of 0.85 and overlapping filters “on”, BCEpred (http://crdd.osdd.net/raghava/bcepred/) predicts epitopes with an 58.7% accuracy using flexibility, hydrophilicity, polarity, and surface properties combined at a threshold of 2.38, and BepiPred 2.0 (http://www.cbs.dtu.dk/services/BepiPred/) predicts B-cell epitopes based on epitopes and nonepitope amino acids determined from crystal structures. Further analysis was performed with IED Antibody Epitope Prediction (Kolaskar and Tongaonkar, 1990).

### 2.5. Three-Dimensional Modelling

The three-dimensional (3D) structure of selected proteins of *A. marginale* and the peroxinectin protein of *R. microplus* were predicted using the PHYRE2 server (Kelley et al., 2015). Phyre2 PDB files were visualized with EzMol tool (Reynolds et al., 2018)

## 3. Results

### 3.1. Protein Selection

We identified 21 conserved amino acid sequences corresponding to membrane-associated proteins in all of the seven Mexican *A. marginale* strains (Table S1, supplementary material). Three protein sequences (PleD, MurJ, and TolC) of *A. marginale* were selected from this list, and the peroxinectin protein sequence of *R. microplus* was chosen after a transcriptomic analysis. The sequences selected have not been previously studied in *A. marginale* and/or *R. microplus* and have attributed functions that make them attractive as potential vaccine candidates.

### 3.2. PleD Family of Two-Component System Response Regulators

PleD is present in all seven Mexican published sequenced strains; it is composed of 455 amino acids, with an approximate molecular weight of 51.4 kDa, and a theoretical pI of 4.99 (Table 1). Clustal Omega alignment showed identity percentages between 99.56% and 100% among all Mexican sequences.

**Figure 1:**
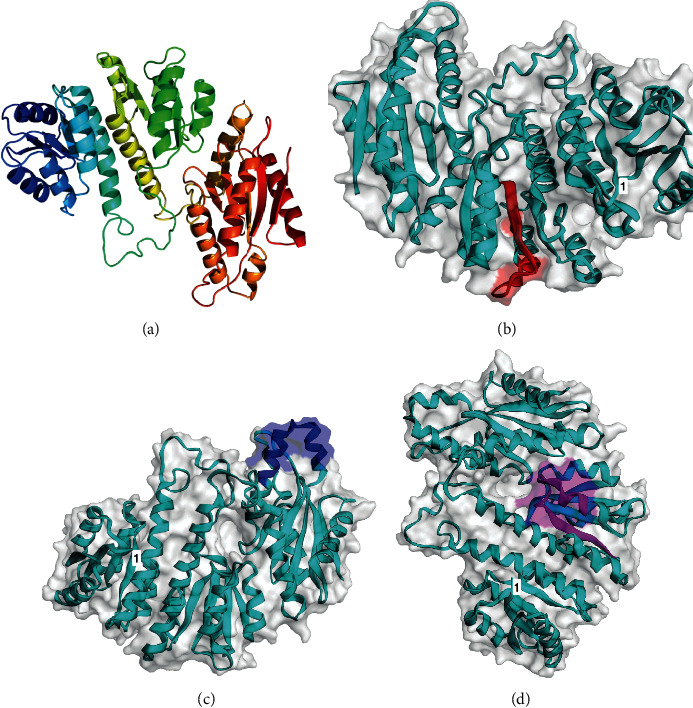
PleD graphic representation. (a) Phyre2 model. (b) SYDLFIIDLNFGGDGLRF epitope (amino acids 200–217); (c) KRVNDTFGHTVGDELLQQ epitope (amino acids 337–354); and (d) NFRSNNNTRYTPILVLLD epitope (amino acids 220–237). These sequences are recognized by all three epitope-prediction programs and have a score above 1.0 when analyzed by Vaxijen. The first residue of the chain is indicated with 1.

The subcellular location explored with CCTOP gave no potential model; thus, the sequence was analyzed with TMHMM, where the proposed model turned out to be an extracellular protein (data not shown), but when analyzed with both the CELLO v.2.5 and the PSORTb servers, they predicted a cytoplasmatic protein, in agreement with the purported function. Functional domain analysis with ScanProsite presents a typical PleD three-domain structure, two of them with the response-regulatory expected domains (4–121 and 158–274 amino acids, respectively) and a GGDEF domain. Vaxijen analysis showed that PleD is a possible antigen at 0.5 threshold. B-cell linear epitope analysis gave several representative sequences. From the analysis with ABCpred at 0.80 threshold and an 18 amino acid-length sequence, there were more than a dozen potential B-cell epitopes, yet only three sequences were recognized by the programs proposed in Materials and Methods. These epitopes were also individually analyzed by Vaxijen, and all three had scores ≥1.0. These sequences are graphically represented in Figure 1 along with the Phyre2 3D model, visualized with the EzMol online tool. The model obtained from Phyre2 analysis was obtained from comparison with the crystal structure of the “Response Regulator PleD” from *Caulobacter vibrioides* (PDB ID: c1w25 B). The 3D model obtained had >90% confidence out of a 41% identity. The three sequences representative of B-cell epitopes are SYDLFIIDLNFGGDGLRF (amino acids 200–217), KRVNDTFGHTVGDELLQQ (amino acids 337–354), and NFRSNNNTRYTPILVLLD (amino acids 220–236). These epitopes are also found in all other *A*. *marginale* sequences available at NCBI.

### 3.3. Murein Biosynthesis Integral Membrane Protein MurJ

**Figure 2:**
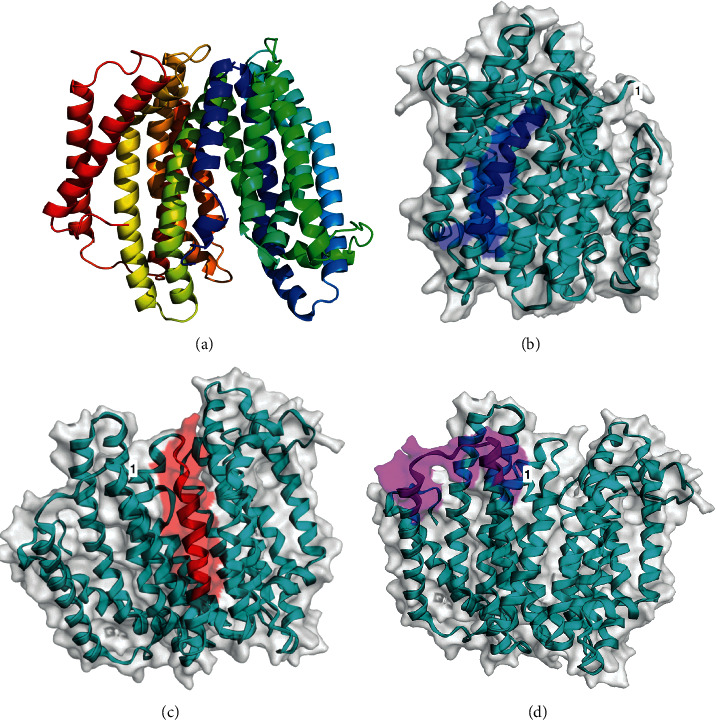
MurJ graphic representation. (a) Phyre2 model; (b) SRIMMVYLFCMSLSSVVC epitope (amino acids 127 to 144); (c) EFKIPAFFSCISVTVNAL epitope (amino acids 373–390); and (d) YLKIHNLYSMSEELSRKL epitope (amino acids 422–439). These sequences are recognized by all three epitope-prediction programs and, when analyzed by Vaxijen, the scores were above 0.75.

MurJ is composed of 501 amino acids with a molecular weight of 54.9 kDa and a theoretical pI of 9.37 (Table 1). Among the sequences of the seven Mexican strains, there are only two amino acid substitutions on positions 151 and 193, with an identity of 99.6 to 100%; thus, it is very conserved. Vaxijen analysis showed that MurJ is not antigenic at 0.5 threshold. B-cell linear epitope analysis gave several representative sequences. We obtained only half a dozen sequences from the analysis with ABCpred at 0.80 threshold and with an amino acid-length of 18, and from these, three sequences were recognized by B-cell epitope-prediction programs. The analysis with Vaxijen reported scores ≥0.75 for SRIMMVYLFCMSLSSVVC (amino acids 127 to 144), EFKIPAFFSCISVTVNAL (amino acids 373–390), and YLKIHNLYSMSEELSRKL (amino acids 422–439). These sequences are present in many other already published works on *A. marginale*, *A. ovis*, and *A. centrale.* The sequences are graphically represented in Figure 2 along with the Phyre2 3D model. Phyre2 analysis also gave a secondary structure model based on the beta sheets, turns, and alpha helix domains, where there are cytoplasmatic amino ends, 14 transmembrane segments, and a cytoplasmatic carboxylic end. Phyre2 program modelled a 3D structure with 100% confidence based on the structure of *E. coli* MurJ, PDB ID : 6CC4 [24].

### 3.4. Outer Membrane Protein TolC

**Figure 3:**
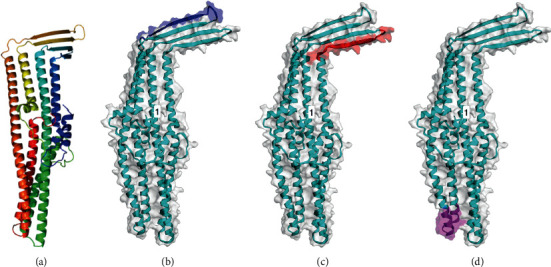
TolC graphic representation. (a) Phyre2 model; (b) INVDKASQRLEVRLRFPV epitope (amino acids 273–290); (c) GFLPRVTYDFVVQKDGRH epitope (amino acids 60–77); and (d) EAIKQEAKLNLKTTLDVL epitope (amino acids 354–371). These sequences were recognized by all three epitope-prediction programs and were as well recognized as probable antigens by Vaxijen with scores above 0.8.

In all Mexican strains, TolC is composed of 408 amino acids, but in the strains of Aguascalientes, Atitalaquia, and Puente de Ixtla, its sequences have a single amino acid change in position 43. The protein has a molecular weight of 45.3 kDa, a theoretical pI of 9.48, and an aliphatic index of 101.25 (Table 1). TMHMM and PSORTb predicted an outer membrane protein, whereas Phyre2 and CCTOP predicted a cytoplasmatic protein. The 3D model obtained from Phyre2, based on the models of its crystallized *E. coli* homolog (PDB ID: c1tqqC) is basically the same as that obtained with CCTOP, except that these tools consider the fact that the TolC final form is a homotrimer [25]. Thus, we consider TolC as a mostly cytoplasmatic protein, with a transmembrane domain that traverses to both internal and external membranes, and a short extracellular domain. Vaxijen analysis shows that at 0.5 threshold, TolC is not antigenic (Table 1). B-cell linear peptides analysis with ABCpred at 0.75 threshold showed about two dozen representative sequences, and from those, only three were chosen for their antigenicity scores, with two other programs. Epitope sequences INVDKASQRLEVRLRFPV (amino acids 273–290), GFLPRVTYDFVVQKDGRH (amino acids 60–77), and EAIKQEAKLNLKTTLDVL (amino acids 354–371) were all recognized as probable antigen by Vaxijen with scores above 0.8. These three epitopes are shown within the context of a 3D model generated with Phyre2 tool and visualized with EzMol (Figure 3). These three B epitope sequences are present in all of the seven Mexican strains and were also present in all other *A. marginale*, *A. centrale*, and *A. ovis* reported genomes.

### 3.5. Peroxinectin of *R. microplus*

In this work, we report, for the first time, a peroxinectin protein from the tick *R. microplus*.

This protein has a molecular weight of 90.35 kDa and a periplasmatic localization according to both CELLO and TMHMM servers (Table 2).

**Figure 4:**
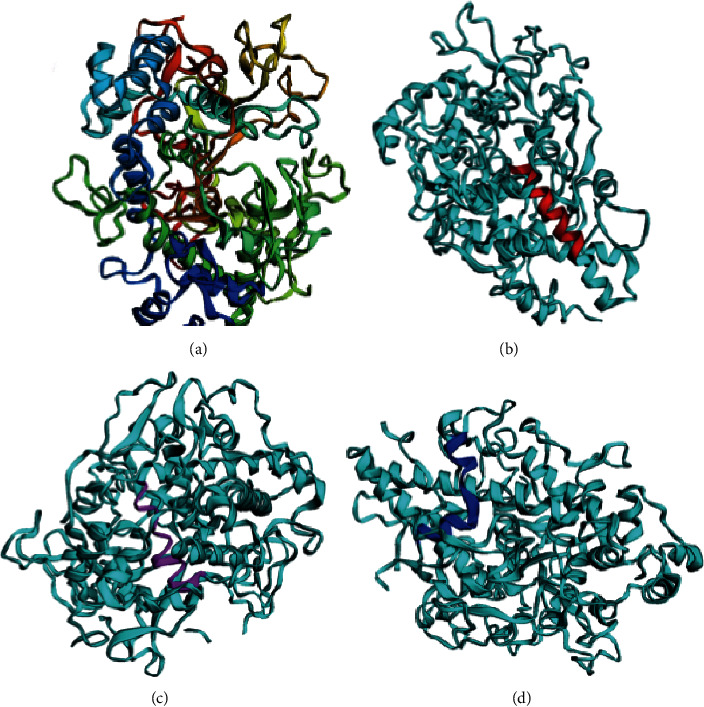
*Rhipicephalus microplus* peroxinectin graphic representation. (a) Phyre2 model; (b) SLTAMHTLWMREHNRV epitope (amino acids 465–481); (c) IGNVFAAAAYRYGHTL epitope (amino acids 550–566); and (d) VEQIRKASLARIICDN, epitope (amino acids 750–766). These sequences were recognized by all three epitope-prediction programs and were visualized with EzMol.

The prediction of ScanProsite shows a peroxidase domain that is shared with chorion peroxonectin of *Ixodes scapularis* with an identity of 94% according to Blastp. Although Vaxijen predicted the peroxinectin as a nonantigenic protein at 0.5 threshold, B-cell linear epitopes predicted with ABCpred at 0.75 threshold showed 45 sequences, and from those, only three were selected (SLTAMHTLWMREHNRV, amino acids 465–481; IGNVFAAAAYRYGHTL, amino acids 550–566; and VEQIRKASLARIICDN, amino acids 750–766) which are shown in the 3D model generated with Phyre2 tool and visualized with EzMol (Figure 4). The 3D model obtained from Phyre2 was based on the model of the crystallized human myeloperoxidase (PDB ID: c5mfaA) with a 100% confidence and a 37% identity with the peroxidase family domain.

## 4. Discussion

Recently, sequencing of genomes and omics approaches for pathogens of veterinary importance and their vectors have provided a significant amount of data. The analysis of these data is an important step in the process of designing new vaccines that can protect against *A. marginale.* The studies of the tick *R. microplus* have contributed to the understanding of the *A. marginale* interactions and the identification of possible targets to control tick infestations [26, 27].

In *A. marginale,* a large number of laboratory studies have focused on the MSPs and the Type Four Secretion System proteins, but none of them have resulted in a vaccine capable of protecting cattle [28, 29, 30]. In the case of *R. microplus*, several recombinant vaccines have been developed; however, the search for new targets continue [6, 8]. In the present work, we have performed an analysis of the complete genome sequences of all Mexican *A. marginale* strains and extracted the sequences of membrane-associated proteins. Our approach was to edit MSPs, TFSS, and OMPs as curated by the NCBI and search for proteins that are highly conserved between all seven strains. From the seven Mexican strains, PleD, MurJ, and TolC were almost identical, with only few amino acid variations, ranging from 99.56% to 99.75% identity (Table 1). The three proteins have important functions in Gram-negative bacteria, and two of them (PleD and TolC) have been studied in other Rickettsiaceae. PleD along with PleC form part of one of the several two-component signal transduction systems in Gram-negative bacteria commonly used to coordinate intracellular responses with environmental cues [31]. MurJ is a lipid II type flippase involved in the translocation of peptidoglycan from the cytoplasm to the periplasmic space in Gram-negative bacteria [32]. While there is no evidence of peptidoglycan synthesis in Rickettsiaceae [33], it seemed reasonable to explore MurJ in this context. Finally, TolC belongs to a family of multidrug transporters that provide an essential first-line defense mechanism against antibiotics, and also for expelling toxic compounds from the cell [34]. There is evidence that, at least, the *R. typhi* ankyrin secretion is dependent on TolC [35]. The role of TolC in *A. marginale* is not known yet, but it has been reported that ankyrin is expressed during the infection of the tick vector, so it is possible that TolC may also play a role during infection of the tick vector. Furthermore, we described, for the first time, the presence of a peroxinectin protein of *R. microplus.* This protein has an important role in immunological processes, including cell adhesion and opsonization. Also, its peroxidase activity has been associated with an efficient microbicidal attack system to invading microorganisms [36]. Ticks infestations propitiate a significant number of bites on cattle, with the concomitant presence of blood and antibodies from the host in the tick. So, we cannot discard the possibility that peroxinectin may be recognized by host antibodies and, then, interfere with its immunological functions, increasing the susceptibility of ticks to external microorganisms that are used in biological control [37]. In arthropods, peroxinectin has an important role in the process of melanization, where melanin acts as a protective barrier as part of a vital mechanism to defense against pathogens [38, 39]. Also, in insects, hemocyte opsonization mediated by peroxinectin facilitates the internalization of bacteria. The activities of peroxinectin reported in insects reinforce the potential of this protein as a probable target to control tick infestations.

The scope of our study was to report potential vaccine candidates under the criterion of conservancy, function, and antigenicity. The three selected proteins of *A. marginale* and the peroxinectin of *R. microplus* fill this criterion. The 3D models obtained allow for the localization of the proposed B-cell epitopes in terms of exposition to possible attack from specific antibodies. Then, peptides that contain these epitopes may be synthetized and used as a new alternative in designing diagnostic targets [40] or for vaccine candidates [41], as well as the use of Multiple Antigenic Peptides (MAPs) where one or even two epitope sequences can be included in a tetramer or an octamer to be synthesized and used as vaccine [41]. In our case, the combination of one or more of these peptides with an antigen from the tick vector may be a better alternative as on one hand, the pathogen is targeted and on the other, the vector can be targeted as well, probably minimizing transmission.

## 5. Conclusions

In this work, we present an alternative to the study of therapeutic targets against pathogens, considering the bioinformatic tools a good strategy for the design of new candidate molecules to control pathogens. Here, we present an alternative collection of immunogenic targets derived from *in silico* analysis of *A. marginale* proteins and their transmission vector *R. microplus.* The B-cell epitopes from the three proteins of *A. marginale*, PleD, MurJ, and TolC, have not been previously reported as immunogenic targets, and they could be a viable alternative to the known proteins used to design vaccines against bovine anaplasmosis. In regards to the *R. microplus* tick, we report the Pxn protein, considered a protein with a role in immunological, cellular, and reproductive mechanisms of tick and, therefore, a good candidate for its control.

## Figures and Tables

**Table 1:** Physicochemical characteristics of *A. marginale* selected proteins.

Protein	PleD	MurJ	TolC
NCBi accession number	KAA8473141	KAA8472909	KAA8472779
Length AA	455	501	408
Minimum % identity	99.56%	99.60%	99.75%
MW Da	51479.39	54909.97	45329.33
Theoretical pI	4.99	9.37	9.48
Aliphatic index	93.01	127.49	101.25
Domains (domain location)	Two response-regulatory (4–121 aa, 158–274 aa) GGDEF domain, (324–455 aa)	Lipid II flippase MurJ (26–484 aa)	Two outer membrane efflux domains, (25–203 aa and 222–403 aa)
Vaxijen threshold	0.5725	0.4482	0.4482
Location	Cytoplasmic 9.97 (PSORTb)	Cytoplasmic 10.00 (PSORTb)	Cytoplasmic protein (CCTOP)

^*∗*^Strain Mex-15 was used as reference for the calculation of parameters of each protein. ^ç^Identity in all cases ranges from 99.56% to 100%

**Table 2:** Physicochemical characteristics of peroxinectin protein.

Length AA	808
MW Da	90352.86
Theoretical pI	6.03
Aliphatic index	78.38
Domains (domain location)	Animal heme peroxidase (228–805 aa)
Vaxijen threshold	0.4894
Location	Periplasmic protein (CELLO, TMHMM)

## Data Availability

Data are available from the corresponding author on request.

## Supplementary Materials

Supplementary Materials Table S1: proteins present in seven Mexican strains of *Anaplasma marginale* with immunogenic properties predicted by *in silico* analysis.

## References

[1] Estrada Peña A., Szabó M., Labruna M. (2019). Towards an effective, rational and sustainable approach for the control of cattle ticks in the neotropics. *Vaccines*.

[2] Quiroz Castañeda R. E., Amaro Estrada I., Rodríguez Camarillo S. D. (2016). *Anaplasma marginale*: diversity, virulence, and vaccine landscape through a genomics approach. *BioMed Research International*.

[3] Aubry P., Geale D. W. (2011). A review of Bovine anaplasmosis. *Transboundary and Emerging Diseases*.

[4] Betancur Hurtado O., Giraldo Ríos C. (2018). *Economic and Health Impact of the Ticks in Production Animals, in: Ticks and Tick-borne Pathogens*.

[5] Camarillo S. D. R., Ortiz M. Á. G., Ramírez E. E. R. (2008). Anaplasma marginale Yucatan (Mexico) Strain: assessment of low virulence and potential use as a live vaccine. *Annals of the New York Academy of Sciences*.

[6] Guerrero F. D., Andreotti R., Bendele K. G. (2014). Rhipicephalus (boophilus) microplus aquaporin as an effective vaccine antigen to protect against cattle tick infestations. *Parasites & Vectors*.

[7] Rego R. O. M., Trentelman J. J. A., Anguita J. (2019). Counterattacking the tick bite: towards a rational design of anti-tick vaccines targeting pathogen transmission. *Parasites & Vectors*.

[8] Trentelman J. J. A., Teunissen H., Kleuskens J. A. G. M. (2019). A combination of antibodies against Bm86 and Subolesin inhibits engorgement of *Rhipicephalus australis* (formerly *Rhipicephalus microplus*) larvae in vitro. *Parasites & Vectors*.

[9] Sarli M., Thompson C. S., Novoa M. B. (2019). Development and evaluation of a double-antigen sandwich ELISA to identify *Anaplasma marginale*–infected and *A. centrale–*vaccinated cattle. *Journal of Veterinary Diagnostic Investigation*.

[10] Zabel T. A., Agusto F. B. (2018). Transmission dynamics of bovine anaplasmosis in a cattle herd. *Interdisciplinary Perspectives on Infectious Diseases*.

[11] Deurenberg R. H., Bathoorn E., Chlebowicz M. A. (2017). Application of next generation sequencing in clinical microbiology and infection prevention. *Journal of Biotechnology*.

[12] Barrero R. A., Guerrero F. D., Black M. (2017). Gene-enriched draft genome of the cattle tick *Rhipicephalus microplus:* assembly by the hybrid Pacific Biosciences/Illumina approach enabled analysis of the highly repetitive genome. *International Journal for Parasitology*.

[13] Brayton K. A., Kappmeyer L. S., Herndon D. R. (2005). Complete genome sequencing of *Anaplasma marginale* reveals that the surface is skewed to two superfamilies of outer membrane proteins. *Proceedings of the National Academy of Sciences*.

[14] Quiroz-Castañeda R. E., Martínez-Ocampo F., Dantán-González E. (2018). Draft genome sequence of mycoplasma wenyonii, a second hemotropic mycoplasma species identified in Mexican bovine cattle. *Microbiology Resource Announcements*.

[15] Macmillan H., Brayton K. A., Palmer G. H. (2006). Analysis of the *Anaplasma marginale* major surface protein 1 complex protein composition by tandem mass spectrometry. *Journal of Bacteriology*.

[16] Torioni de Echaide S., Knowles D. P., McGuire T. C., Palmer G. H., Suarez C. E., McElwain T. F. (1998). Detection of cattle naturally infected with *Anaplasma marginale* in a region of endemicity by nested PCR and a competitive enzyme-linked immunosorbent assay using recombinant major surface protein 5. *Journal of Clinical Microbiology*.

[17] Willadsen P. (2008). *Anti-tick Vaccines*.

[18] Willadsen P., Bird P., Cobon G. S., Hungerford J. (1995). Commercialisation of a recombinant vaccine against *Boophilus microplus*. *Parasitology*.

[19] Rodríguez M., Penichet M. L., Mouris A. E. (1995). Control of *Boophilus microplus* populations in grazing cattle vaccinated with a recombinant Bm86 antigen preparation. *Veterinary Parasitology*.

[20] Andreotti R., Pérez de León A. A., Dowd S. E., Guerrero F. D., Bendele K. G., Scoles G. A. (2011). Assessment of bacterial diversity in the cattle tick *Rhipicephalus (Boophilus) microplus* through tag-encoded pyrosequencing. *BMC Microbiology*.

[21] Fuente J. d. l., Almazán C., Canales M., Pérez de la Lastra J. M., Kocan K. M., Willadsen P. (2007). A ten-year review of commercial vaccine performance for control of tick infestations on cattle. *Animal Health Research Reviews*.

[22] Aguilar Díaz H., Esquivel Velázquez M., Quiroz Castañeda R. E. (2018). Comparative hemolymph proteomic and enzymatic analyses of two strains of *Rhipicephalus (boophilus) microplus* ticks resistant and susceptible to ixodicides. *BioMed Research International*.

[23] Maruyama S. R., Garcia G. R., Teixeira F. R. (2017). Mining a differential sialotranscriptome of *Rhipicephalus microplus* guides antigen discovery to formulate a vaccine that reduces tick infestations. *Parasites & Vectors*.

[24] Zheng S., Sham L.-T., Rubino F. A. (2018). Structure and mutagenic analysis of the lipid II flippase MurJ from escherichia coli. *Proceedings of the National Academy of Sciences*.

[25] Jeong H., Kim J.-S., Song S. (2016). Pseudoatomic structure of the tripartite multidrug efflux pump AcrAB-TolC reveals the intermeshing cogwheel-like interaction between AcrA and TolC. *Structure*.

[26] Antunes S., Couto J., Ferrolho J. (2019). Transcriptome and proteome response of rhipicephalus annulatus tick vector to babesia bigemina infection. *Frontiers in Physiology*.

[27] Kules J., Horvatic A., Guillemin N., Galan A., Mrljak V., Bhide M. (2016). New approaches and omics tools for mining of vaccine candidates against vector-borne diseases. *Molecular BioSystems*.

[28] Brown W. C., Barbet A. F. (2016). Persistent infections and immunity in ruminants to arthropod-borne bacteria in the family anaplasmataceae. *Annual Review of Animal Biosciences*.

[29] Lockwood S., Voth D. E., Brayton K. a. (2011). Identification of *Anaplasma marginale* type IV secretion system effector proteins. *PLoS One*.

[30] Pruneau L., Moumène A., Meyer D. F., Marcelino I., Lefrançois T., Vachiéry N. (2014). Understanding anaplasmataceae pathogenesis using “omics” approaches. *Frontiers in Cellular and Infection Microbiology Microbiol.*.

[31] Stock A. M., Robinson V. L., Goudreau P. N. (2000). Two-component signal transduction. *Annual Review of Biochemistry*.

[32] Palenzuela N. (2008). Bioinformatics identification of MurJ (MviN) as the peptidoglycan lipid II flippase in escherichia coli. *Proceedings of the National Academy of Sciences*.

[33] Lin M., Rikihisa Y. (2003). *Ehrlichia chaffeensis* and *Anaplasma phagocytophilum* lack genes for lipid a biosynthesis and incorporate cholesterol for their survival. *Infection and Immunity*.

[34] Schuldiner S. (2018). The *escherichia coli* effluxome. *Research in Microbiology*.

[35] Kaur S. J., Rahman M. S., Ammerman N. C. (2012). TolC-dependent secretion of an ankyrin repeat-containing protein of *Rickettsia typhi*. *Journal of Bacteriology*.

[36] Holmblad T., Söderhäll K. (1999). Cell adhesion molecules and antioxidative enzymes in a crustacean, possible role in immunity. *Aquaculture*.

[37] Samish M., Ginsberg H., Glazer I. (2004). Biological control of ticks. *Parasitology*.

[38] Bilandžija H., Laslo M., Porter M. L., Fong D. W. (2017). Melanization in response to wounding is ancestral in arthropods and conserved in albino cave species. *Scientific Reports*.

[39] Thörnqvist P.-O., Johansson M. W., Söderhäll K. (1994). Opsonic activity of cell adhesion proteins and *β*-1,3-glucan binding proteins from two crustaceans. *Developmental & Comparative Immunology*.

[40] Quiroz Castañeda R. E., Tapia Uriza T. R., Valencia Mujica C. (2019). Synthetic peptides-based indirect ELISA for the diagnosis of bovine anaplasmosis. *International Journal of Applied Research in Veterinary Medicine*.

[41] Blanco E., Guerra B., de la Torre B. G. (2016). Full protection of swine against foot-and-mouth disease by a bivalent B-cell epitope dendrimer peptide. *Antiviral Research*.

